# The long-term health consequences of genocide: developing GESQUQ - a genocide studies checklist

**DOI:** 10.1186/s13031-019-0198-9

**Published:** 2019-04-11

**Authors:** Jutta Lindert, Ichiro Kawachi, Haim Y. Knobler, Moshe Z. Abramowitz, Sandro Galea, Bayard Roberts, Richard Mollica, Martin McKee

**Affiliations:** 1 Department of Health and Social Work, University of Applied Sciences Emden/Leer, Constantiaplatz 4, 26723 Emden, Germany; 2 Women’s Research Center, Brandeis University, Waltham, Massachusetts USA; 3 Department of Social and Behavioral Sciences, Harvard T.H. Chan School of Public Health, Boston, Massachusetts USA; 4 Hadassah Medical School, Jerusalem, Israel; 5 Dean’s Office, Boston University School of Public Health, Boston, Massachusetts USA; 6 Department of Health Services Research and Policy, London School of Hygiene and Tropical Medicine, London, UK; 7 Harvard Program in Refugee Trauma, Department of Psychiatry, Massachusetts General Hospital, Harvard Medical School, Cambridge, USA

**Keywords:** Genocide, Long term impact, Quality criteria, Mental health, Bias

## Abstract

**Background:**

Genocide is an atrocity that seeks to destroy whole populations, leaving empty countries, empty spaces and empty memories, but also a large health burden among survivors is enormous. We propose a genocide reporting checklist to encourage consistent high quality in studies designed to provide robust and reliable data on the long term impact of genocide.

**Methods:**

An interdisciplinary (Public Health, epidemiology, psychiatry, medicine, sociology, genocide studies) and international working committee of experts from Germany, Israel, the United States, and the United Kingdom used an iterative consensus process to develop a genocide studies checklist for studies of the long term health consequences.

**Results:**

We created a list of eight domains (A Ethical approval, B External validity, C Misclassification, D Study design, E Confounder, F Data collection, G Withdrawal) with 1–3 specific items (total 17).

**Conclusion:**

The genocide studies checklist is easy to use for authors, journal editors, peer reviewers, and others involved in documenting the health consequences of genocide.

**Electronic supplementary material:**

The online version of this article (10.1186/s13031-019-0198-9) contains supplementary material, which is available to authorized users.

## Background

Genocides have brought immeasurable suffering to millions of people in the 20th and the early years of the twenty-first century. [1, 2] They have attracted the attention of researchers from a range of disciplines, including epidemiologists, historians, political scientists, psychologists, anthropologists, demographers, and others, with genocide studies emerging as a distinct body of scholarship. Each discipline offers important perspectives on a phenomenon whose horror is beyond the imagination of most people. The impact of genocide continues long after the killing has ended, leaving lifelong scars on survivors and, potentially, their offspring. [2] Yet, as revealed in a recent systematic review, this research has taken a wide variety of approaches and produced heterogeneous, and, in some cases, conflicting findings, suggesting a range of consequences between severe long term impact and no impact. [2] This conflicting evidence leads to several conclusions.

First, genocide - as defined in the Convention on the Prevention and Punishment of the Crime of Genocide (CPPCG) adopted by the United Nations General Assembly on 9th of December 1948 as General Assembly Resolution 260 (III, article 2) - can take many forms, from the semi-organized chaos of Rwanda systematic murder of Jews by Nazis and/or their allies in the Genocide termed the Holocaust, with differences in exposures to mass atrocities (e.g. duration, types of genocidal acts). The Convention defines genocide as an attempt “to destroy, in whole or in part, a national, ethnical, racial or religious group.” Genocidal acts include “killing members of the group; [and] causing serious bodily or mental harm to members of the group”, and deliberately inflicting “conditions of life, calculated to bring about [a group’s] physical destruction in whole or in part; imposing measures intended to prevent births within the group; [and] forcibly transferring children of the group to another group”. [3] These definitions suggest a breadth of exposures that can be associated with genocide. Table 1 lists those events that have been designated officially by the United Nations as genocides, as well as the range of estimates of those killed and the percentages of the target populations affected.

**Table 1:** Genocides since World War 1

Event	Country	Start (year)	End (year)	Lowest estimate	Highest estimate	% affected
Herero and Namqua genocide	German South-West Africa	1904	1908	34,000	110,000	No information
Seyfo	Ottoman Empire	1915	1923	275,000	750,000	No information
Armenian genocide	Ottoman Empire	1915	1922	800,000	1,500,000	75% of Armenians in Turkey
Holocaust	Nazi- controlled Europe	1933	1945	4,900,000	6,200,000	78% of Jews in Nazi-controlled Europe
Porajmos	Nazi- controlled Europe	1935	1945	130,000	500,000	25% of Romani people in Europe
Cambodian genocide	Cambodia	1975	1979	1,700,000	3,000,000	21–33% of total population of Cambodia
Bosnian genocide	Bosnia and Herzegovina	1992	1995	9373	25,609	No information
Rwandan genocide	Rwanda	1994	1994	500,000	1,000,000	70% of Tutsis in Rwanda
Genocide of Yazidis	Iraq and Syria	2014	ongoing	?	?	No information

Source: authors’ compilation

A second concern relates to methodological differences among studies. For example, studies reviewing the mental health impact of genocides have investigated a variety of outcomes, including depression, anxiety, schizophrenia [4, 5], suicide [6, 7], post-traumatic stress as well post-traumatic growth. Some studies documented a negative impact, while others found resilience or no association notwithstanding immense cruelties to which survivors had been exposed. [8] Some of this variability may be due to the methodological challenges inherent in conducting studies on populations affected by genocide. Some are common to any epidemiological research and include recruitment bias, measurement error, and the need to adjustment for potential confounding. Attempts to attribute symptoms to the experience of genocide may be complicated by confounding factors unrelated to the genocide, such as discrimination in another country due to migration or poverty. [9] Other factors, however, are specific to genocide research. One is memoralization, whereby groups valorize, marginalize, or disable acts of remembrance, or forgetting. [10, 11] Anthropological research has reported how some genocide survivors or children of survivors challenge the pathologizing construct of long term impact of genocides. It can be politically expedient to pathologize the long term consequences of genocide, or, conversely, to deny the long term impacts of genocides as part of an attempt to relieve the perpetrator from responsibility for having committed genocide. Disorders associated with genocide are therefore subject to the influence of various interests, institutions, and political interests.

The need for clear, transparent, and reliable reporting of research has led to important initiatives such as the Strengthening Reporting of Observational Studies in Epidemiology Statement (STROBE). [12] The STROBE statement, published in 2007, is an evidence-based 22 item set of recommendations for reporting observational studies (cohort studies, case-control studies, and cross-sectional studies) and has been credited with improvements in quality of reporting. [13] However, the STROBE statement is designed to apply to all observational studies, [8] and it does not adequately capture some of the key challenges inherent in post-genocide research.

Seeking to address this shortcoming, an international group of experts (JL, MZA, HJK, SG, RM, BR, MMcK, IK) with a specific interest in genocide and health worked together on a systematic review. [2] Important gaps in STROBE that were specific to studies of genocide and health were identified and agreement was reached that an extension of STROBE was warranted. Thus, the QUALITY ASSESSMENT TOOL FOR QUANTITATIVE GENOCIDE STUDIES (GESUQ) initiative was established as an international collaborative project to address these issues. Herein, we propose recommendations for reporting genocide and related research.

## Methods

First, we searched for any existing reporting guideline covering long term impacts of genocide. Second, we sought relevant evidence regarding the quality of reporting. Neither search yielded any results. Third, we identified experts (i.e., methodologists, psychiatrists, epidemiologists, and genocide experts) who could advise on potential sources of bias, from relevant genocide projects and reference documents. They were then asked for recommendations. Fourth, the group met in person and via Skype meetings to agree the wording of the statements. Stakeholders reviewed the statements and provided feedback. The final checklist and this explanatory document were drafted by the three members of the working committee. During two face-to-face and three skype meetings, members of the group discussed the input received and prepared new versions that were circulated until consensus on all items was reached.

## Results

### Items in the GESUQ checklist

The complete GESUQ checklist is provided in the Additional file 1. In the following sections we explain the rationale for choosing items A-H in GESUQ.

#### A. Ethical approval

Research on the impact of genocide must adhere to same ethical standards that guide all other research. [14] This includes that all research participants have the capacity to provide informed consent. As in other research, investigators should maintain the principles of approval of research by institutional review boards (IRBs) respecting not only confidentiality and privacy but the importance of expertise in genocide research within the research team, including the specific genocide-related challenges that exist. Among these challenges are extra provisions to minimize harm to human subjects (e.g. the potential for retaliation from those who perpetrated the genocide), and extra steps to provide medical resources / referral to people still suffering from lingering mental health effects. Given the particular risk of causing distress by asking questions about past events in this vulnerable population there is a need to ensure mechanisms for referral information for mental health support. These ethical questions are especially difficult in situations in which genocide perpetrators, genocide victims, and genocide bystanders are forced to live together even after the genocide (e.g. in the case of Rwanda [15]).

#### B. External validity and selection bias

In genocide studies as in other epidemiological studies, attention to sampling methods is crucial. Often in the early aftermath of genocide, health studies either comprise only convenience samples or clinical samples (populations that manifest some kind of pathology, i.e. post-traumatic symptoms) and have sought and obtained care. This is understandable given the challenges of recruitment but is likely to introduce bias as such participants go through several stages of selection and thus, both in practice and theory, may differ from participants drawn from random samples of those exposed. Random sampling should therefore be used. Where this is not possible, analyses should include appropriate weighting. This can avoid the challenge of biased estimates of the incidence and prevalence of certain disorders (e.g., Posttraumatic Stress Disorder). Given the difficulty of random sampling in many settings, alternative methods such as respondent driven sampling may be useful adjuncts. [16]

It is important to recognize the inevitable scope for survivor bias, both in terms of surviving the events in question and their sequelae. Consequently, and to a greater degree with genocide than many other exposures, any sample will not be representative of all those exposed.

#### C. Avoiding misclassification

Any flaw in measuring exposure, outcome, or covariates can overestimate or underestimate the true value of the association. [17–19] This is a challenge in many areas of epidemiology, but is especially so with genocide. Does exposure include direct and indirect exposure (such as the death of family members or friends, and if so, in the presence or absence of the subject)? How is the duration of exposure measured? Reporting of genocide exposure should include the nature, intensity, and length of exposure. This assessment of exposure could draw on approaches adopted in other areas of epidemiology, such as the job-exposure matrix used in occupational epidemiology. [20, 21] Accordingly, assessment of exposure should be quantified systematically. For example, one could inquire about direct personal experiences of genocide (e.g. whether one’s relatives were killed). But in genocide research it is also relevant to assess exposure to genocide even if someone was not directly affected - i.e. there may be spillover effects of genocide in a community. [22] In genocide research both direct and indirect exposures are of interest. Research on the impact of genocide on subsequent generations creates additional challenges, in measuring both the nature and timing of exposure. [2, 5] Our guidelines seek to guide researchers to be explicit about why the exposure measurement was carried out in a certain way.

Genocide studies seek accuracy in reporting the incidence, prevalence, and burden of disease so avoiding diagnostic errors is crucial. It is essential to understand the psychometric properties of health measures used among those affected by genocide. Expressions of suffering due to genocide may differ by populations. In the area of mental health, the DSM-5 [23] emphasizes the need for measures that capture culturally grounded concepts of distress, [24] something that is largely missing from genocide studies so far.

#### D. Study design

Most genocide studies will, inevitably, be retrospective and observational, e.g. case-control or cohort studies. The selection of controls is a major challenge as they should resemble, as closely as possible, those who experienced the genocide without themselves being exposed. The objective of genocide studies therefore is to find an unexposed control group that resembles the exposed group as closely as possible. For example, in studies of the health effects of the Holocaust, investigators have compared Jews who emigrated to Israel before and after the Holocaust. However, even this design poses challenges, since there will be many unobserved factors that could confound the comparison being made, e.g. those who escaped before the Holocaust may have had more extensive social networks to help them escape, and stronger social networks would make such individuals more resilient to adverse mental health effects.

Research undertaken in Israel has used the National Population Register. This is a unique resource for genocide studies. [4] However, should such a situation arise in the future, the ability to use a similar resource will depend upon the nature of consent given at enrolment, the degree of anonymisation, and the data protection laws in place in the country concerned.

#### F. Confounders

If the question of interest involves identifying the causal effects of genocide experience on mental health outcomes, the investigator must identify (and control for) factors that influence the probability of both the exposure and the outcome being studied. For example, in a study of exposure to genocide and the outcome of poor mental health, socioeconomic disadvantage could be a confounder. Someone disadvantaged in this way may be less able to escape the that would make a person less able to escape the direct and indirect effects of genocide, for example because of fewer material resources. However, there is also a well-recognized association between disadvantage and adverse mental health outcomes. It is good epidemiological practice to control for confounders but it is also important not to over control. Thus, genocide is often the final end-result of many decades (perhaps even centuries) of unjust treatment of a particular group in society. Hence, a confounder such as “low socioeconomic status” may itself represent an effect of the underlying injustice which preceded genocide. For example, immediately before and during the Holocaust, many Jewish children and adolescents were excluded from studies in public schools. One would not control for “confounding” by educational attainment in this instance, because interruption of schooling is part of the exposure (the Holocaust) that we are trying to understand. Likewise, the experience of escaping from the genocide constitutes a part of the exposure.

There are several approaches to addressing these issues, including the thorough review of the literature and use of methods such as directed acyclic graphs (DAGs), [25] multivariable regression to help adjust for potential confounders and structural equation modelling (path analysis). [26]

#### F. Data collection methods

Data can be collected in ad hoc surveys or, in rare cases, as with the Israeli National Population Register, from routine sources, as noted above. Valid and reliable assessment of exposures and outcomes requires carefully developed instruments, which have ideally been evaluated in different cultural settings. The attributes (e.g. recall period, question/response format) and mode of administration (e.g. interviewer-, self-administered) of existing instruments are extremely varied and many have not been evaluated for use in different cultural contexts or age groups.

## Discussion

In summary, there are substantial challenges in epidemiological studies of survivors of mass atrocities, crimes against humanity, and genocide. However, data are needed to better serve this population. The GESUQ guidelines are a first step to better understand the mental health impact of mass atrocities, crimes against humanity, and genocide. These proposed guidelines are specific to observational genocide research and serve as starting point for improving epidemiological research on the impact of violence on health. GESUQ was created as a guide for authors, journal editors, peer reviewers, and other stakeholders to encourage researchers to improve the quality and completeness of reporting in genocide and war epidemiology. To our knowledge, our guidelines are the first to have been proposed for use specifically in genocide studies. As with other reporting guidelines, these complement the instructions in editorial and review processes to ensure a clear and transparent account of the research conducted. Experts we contacted generally welcomed the initiative and provided constructive feedback. The checklist will subsequently be translated into other languages, and disseminated widely. Ongoing feedback is encouraged to improve it.

While GESUQ represents our best attempt to reflect the interest and priorities of stakeholders and the interested public in genocide research, we recognize that the methods used in observational health research are changing rapidly, and the availability of different types of data for such research is expanding. For example, mobile health applications (mHealth apps) are becoming widely available for smartphones and wearable technologies. While there is limited experience so far with these data sources in genocide research, we anticipate rapid growth in the near future. Additionally, health care providers in for instance Israel offer good registry data on more outcomes than government data that can be linked to Holocaust exposure without sample selection.

The nature of genocide poses some obvious limitations on the conduct of associated research. First, while we have described this instrument as one for use in genocide studies, the international community has shown a great reluctance to use the term “genocide” because of the implications, in particular the “responsibility to protect”. However, the issues we have discussed in developing this instrument will be equally applicable in many situations involving large scale killings that are not subsequently labelled as genocide. Second, it will continue to be extremely difficult to collect data in real time and any attempt to do so would face a myriad of methodological and ethical challenges, as was apparent in studies seeking to quantify the death toll in post-invasion Iraq. Consideration of these issues goes beyond the scope of this paper.

## Conclusions

We have created GESUQ in the form of a checklist, trying to take account of and learn from existing guidelines. While we anticipate that GESUQ will change as research methods evolve, these guidelines should encourage better reporting of research over the coming years. With implementation by authors, journal editors, and peer reviewers, we anticipate that GESUQ will improve transparency, reproducibility, and completeness of reporting of research on genocide and health and, especially, much-needed research on evidence-based interventions for genocide affected populations.

## Additional file

Additional file 1: Quality assessment tool for quantitative genocide studies. (DOCX 32 kb)

## Abbreviations

BR: Prof. Bayard Roberts
CPPCG: Convention on the Prevention and Punishment of the Crime of Genocide
DAGs: Directed acyclic graphs
GESUQ: Quality Assessment Tool for Quantitative Genocide Studies
HYK: Prof. Haim Y. Knobler
IK: Prof. Ichiro Kawachi
IRBs: Institutional review boards
JL: Prof. Jutta Lindert
mHealth apps: Mobile health applications
MMK: Prof. Martin McKee
MZA: Dr. Moshe Z. Abramowitz
RM: Prof. Richard Mollica
SES: Socioeconomic status
SG: Prof. Sandro Galea
STROBE: Strengthening Reporting of Observational Studies in Epidemiology Statement
