# Supplementary material for: The long-term health consequences of genocide: developing GESQUQ - a genocide studies checklist
Source: Confl Health. 2019 Apr 11;13:14. doi: 10.1186/s13031-019-0198-9 (PMC6460659; doi:10.1186/s13031-019-0198-9)
Supplement: Supplementary file 1 — Quality assessment tool for quantitative genocide studies. (DOCX 32 kb) [file 13031_2019_198_MOESM1_ESM.docx]

**Additional file 1**

**Table 1: QUALITY ASSESSMENT TOOL FOR QUANTITATIVE GENOCIDE STUDIES**

**Adapted and modified from 1) the Effective Public Health Practice Project Quality Assessment Tool for Quantitative Studies, 2) Guidelines for evaluating prevalence studies in mental health.**

**Article ID**

**Journal of publication:**

**Year of publication:**

**Name of first author:**

**COMPONENT RATINGS**

1. **ETHICAL APPROVAL OF THE STUDY**

| A | Not reported | Poor reporting |
| --- | --- | --- |
| B | Study was approved by an ethical committee | Strong |

| RATE THIS SECTION | STRONG | MODERATE | WEAK |
| --- | --- | --- | --- |
| Overall | 1 | 2 | 3 |

1. **EXTERNAL VALIDITY AND SELECTION BIAS**

| (Q1) Sampling of the subjects by the investigators | | |
| --- | --- | --- |
| A | Not reported | Poor reporting |
| B | Randomized population sample based on population registries | Strong |
| C | Whole populations | Strong |
| D | Random sample restricted to geographic area | Moderate |
| E | Convenience sample | Weak |
| F | Purposive sample of survivors groups | Weak |
| G | Health and / or psychiatric care based | Weak |
| H | Medical records based sample | Weak |
| I | Cannot tell | Weak |

| (Q2) Assessment of estimate bias: What percentage of the population approached agreed to participate? | | |
| --- | --- | --- |
| A | Not reported | Poor reporting |
| B | 80-100% agreement | Strong |
| C | 60-79% agreement | Strong |
| D | Less than 60% agreement | Moderate |
| E | Less than 50% Agreement | Weak |

| (Q3) Assessment of sampling bias | | |
| --- | --- | --- |
| A | No information about sampling bias | Poor reporting |
| B | Sampling bias was assessed by the authors; differences in study population vs. target population are reported | Strong |
| C | The authors did not assess sampling bias | Weak |
| D | Other: specify___ |  |

| RATE THIS SECTION | STRONG | MODERATE | WEAK |
| --- | --- | --- | --- |
| Overall | 1 | 2 | 3 |

1. **MISCLASSIFICATION BIAS**

| (Q1) Was the exposure clearly defined for the genocide group? | | |
| --- | --- | --- |
| A | Not reported | Poor reporting |
| B | Specific characteristics of the genocide as exposure were clearly defined | Strong |
| C | Genocide as exposure | Moderate |
| D | No clear distinction between non-exposed and exposed group | Weak |

| RATE THIS SECTION | STRONG | MODERATE | WEAK |
| --- | --- | --- | --- |
| Overall | 1 | 2 | 3 |

1. **STUDY DESIGN**

| (Q1) Indicate the study design | | |
| --- | --- | --- |
| A | Cohort (exposed and not exposed) | Strong |
| B | Case-control | Moderate |
| C | Cross sectional | Weak |
| D | Other: specify___ |  |

| (Q2) Are the controls selected in an appropriate way? | | |
| --- | --- | --- |
| A | Controls are matched | Strong |
| B | Controls without matching | Moderate |
| C | Rationale for selecting controls not reported | Weak |

| RATE THIS SECTION | STRONG | MODERATE | WEAK |
| --- | --- | --- | --- |
| Overall | 1 | 2 | 3 |

1. **CONFOUNDER**

| (Q1) Were there important uncontrolled differences in the genocide-exposed group and in the control group? | | |
| --- | --- | --- |
| A | No important differences between genocide and control group | Strong |
| B | Differences between genocide and control group mentioned in the limitations | Moderate |
| C | Differences between genocide and control group not mentioned | Weak |

| (Q2) Were potential confounders investigated? | | |
| --- | --- | --- |
| A | Yes, life events and socio-demographics were investigated | Strong |
| B | Yes socio-demographics investigated | Moderate |
| C | Other potential confounders were investigated | Moderate |
| D | No confounders investigated | Weak |

| (Q3) If yes, indicate the percentage of relevant confounders that were controlled (either in design (e.g. stratification, matching) or analysis? | | |
| --- | --- | --- |
| A | Life events and socioeconomic status (SES) controlled for | Strong |
| B | Life events or SES controlled for | Moderate |
| C | Other factors controlled for | Moderate |
| D | No other factors | Weak |

| RATE THIS SECTION | STRONG | MODERATE | WEAK |
| --- | --- | --- | --- |
| Overall | 1 | 2 | 3 |

**The following are examples of potential confounders in genocide studies**

1. Gender
2. Marital status
3. Age at exposure
4. Education
5. Socio-economic status (income or class)
6. Genocide characteristics
7. Migration of survivors
8. Discrimination of survivors after the genocide
9. Health status, e.g. substance abuse
10. Prior or current psychotherapy / counselling
11. Life events after the genocide
12. **DATA COLLECTION METHODS**

| A | Not reported | Poor reporting |
| --- | --- | --- |
| B | Measured with validated instruments (norm values available) | Strong |
| C | Measured with newly developed instruments | Moderate |
| D | Standardized clinical interviews | Moderate |
| E | Unstandardized clinical interviews | Weak |
| F | Obtained from medical records | Weak |
| G | Obtained from administrative database | Weak |
| H | Obtained from registries | Weak |
| I | Other | Weak |

| RATE THIS SECTION | STRONG | MODERATE | WEAK |
| --- | --- | --- | --- |
| Overall | 1 | 2 | 3 |

1. **WITHDRAWALS AND DROP-OUTS**

| (Q1) Were withdrawals and drop-outs reported in terms of numbers and / or reasons per group? | | |
| --- | --- | --- |
| A | Withdrawals and drop outs were reported | Strong |
| B | Withdrawals and drop outs were not reported | Weak |
| C | Not applicable (i.e. one time survey or interviews) | Weak |

| (Q2) Indicate the percentage of participants completing the study. (If the percentage differs by groups, record the lowest) | | |
| --- | --- | --- |
| A | 80-100% | Strong |
| B | 60-79% | Moderate |
| C | Less than 60% | Weak |
| D | Not reported | Weak |

| (Q3) Is a power calculation reported? | | |
| --- | --- | --- |
| A | Yes | Strong |
| B | No | Weak |

| RATE THIS SECTION | STRONG | MODERATE | WEAK |
| --- | --- | --- | --- |
| Overall | 1 | 2 | 3 |

1. **ANALYSES**

| (Q1) Are the statistical methods appropriate for the study design? | | |
| --- | --- | --- |
| A | Yes | Strong |
| B | Yes, reasonably well | Moderate |
| C | No | Weak |

| (Q2) Were confidence intervals reported? | | |
| --- | --- | --- |
| A | Yes | Strong |
| B | No | Weak |

| (Q3) Were analyses of subgroups (e.g. according to level of exposure, gender) done? | | |
| --- | --- | --- |
| A | Yes | Strong |
| B | Yes, reasonably well | Moderate |
| C | No | Weak |

| RATE THIS SECTION | STRONG | MODERATE | WEAK |
| --- | --- | --- | --- |
| Overall | 1 | 2 | 3 |

**GLOBAL RATING FOR THIS PAPER (circle one):**

STRONG (one WEAK rating)

MODERATE (two WEAK ratings)

WEAK (three or more WEAK ratings)

VERY WEAK (four or more WEAK ratings)

With all reviewers discussing the ratings:

**Is there a discrepancy between the two reviewers with respect to the component (A-H) ratings?**

1. No
2. Yes

**If yes, indicate the reason for the discrepancy**

1. Oversight
2. Differences in interpretation of criteria
3. Differences in interpretation of study

**Final decision of both reviewers (circle one):**

1. STRONG
2. MODERATE
3. WEAK
4. VERY WEAK
